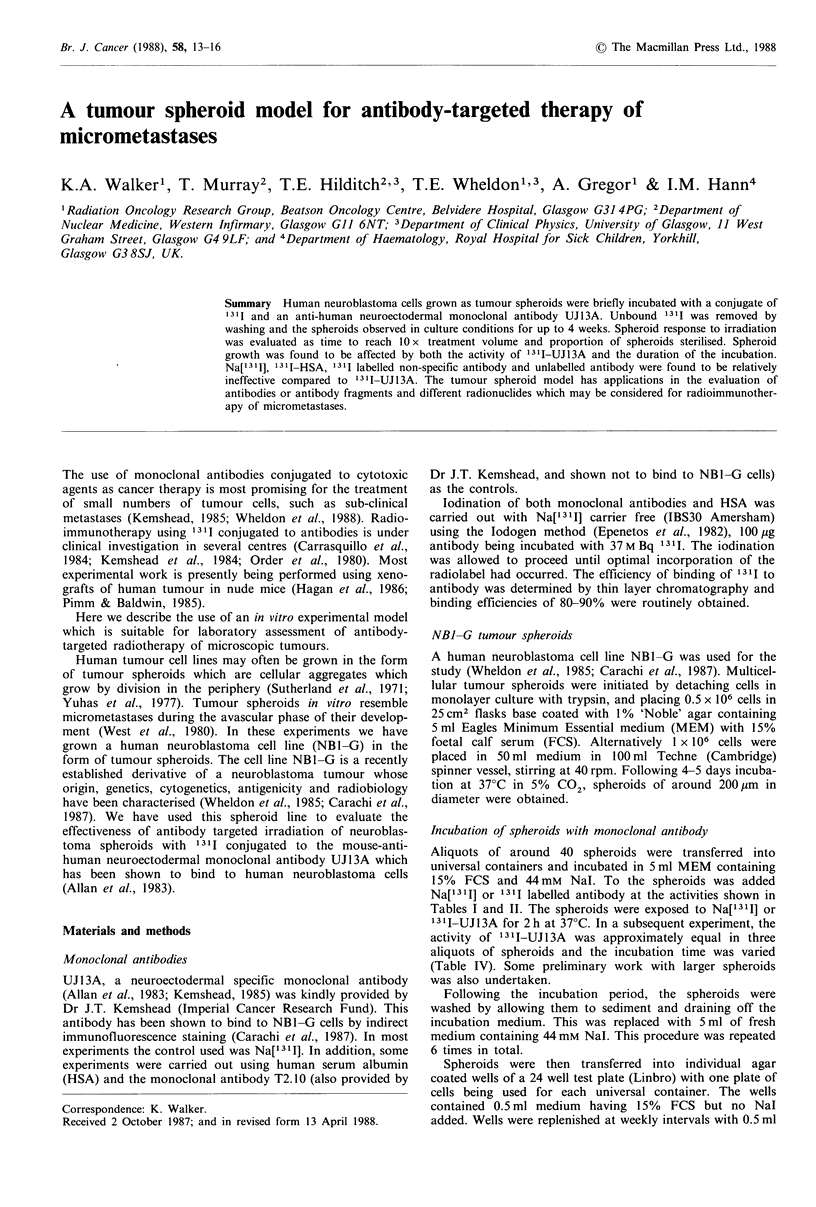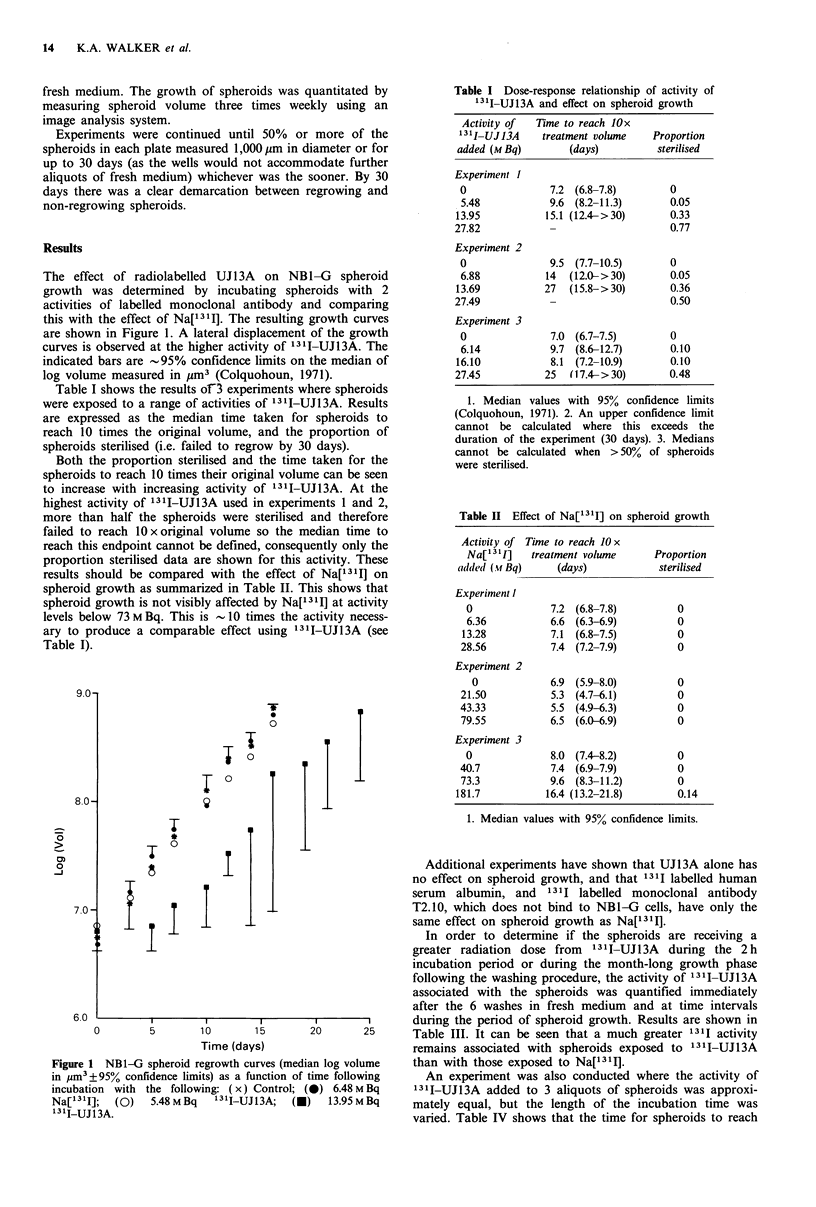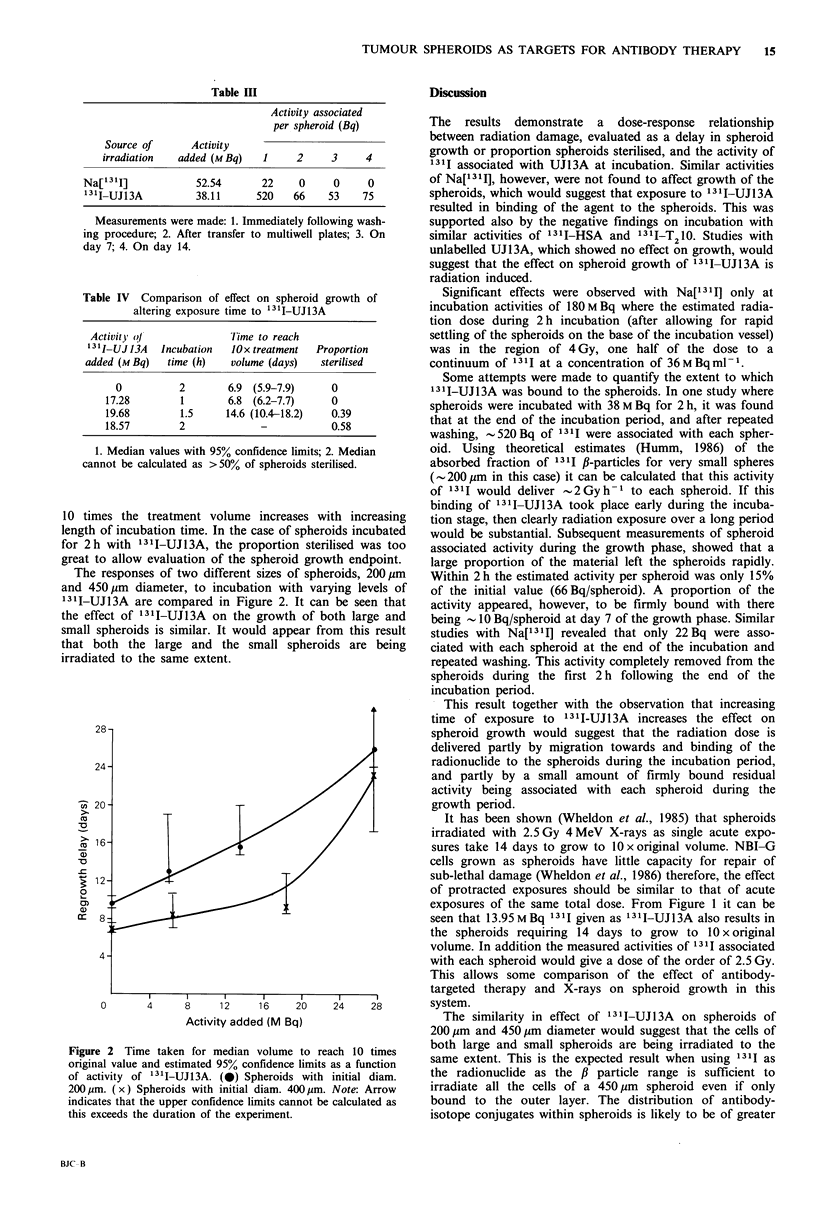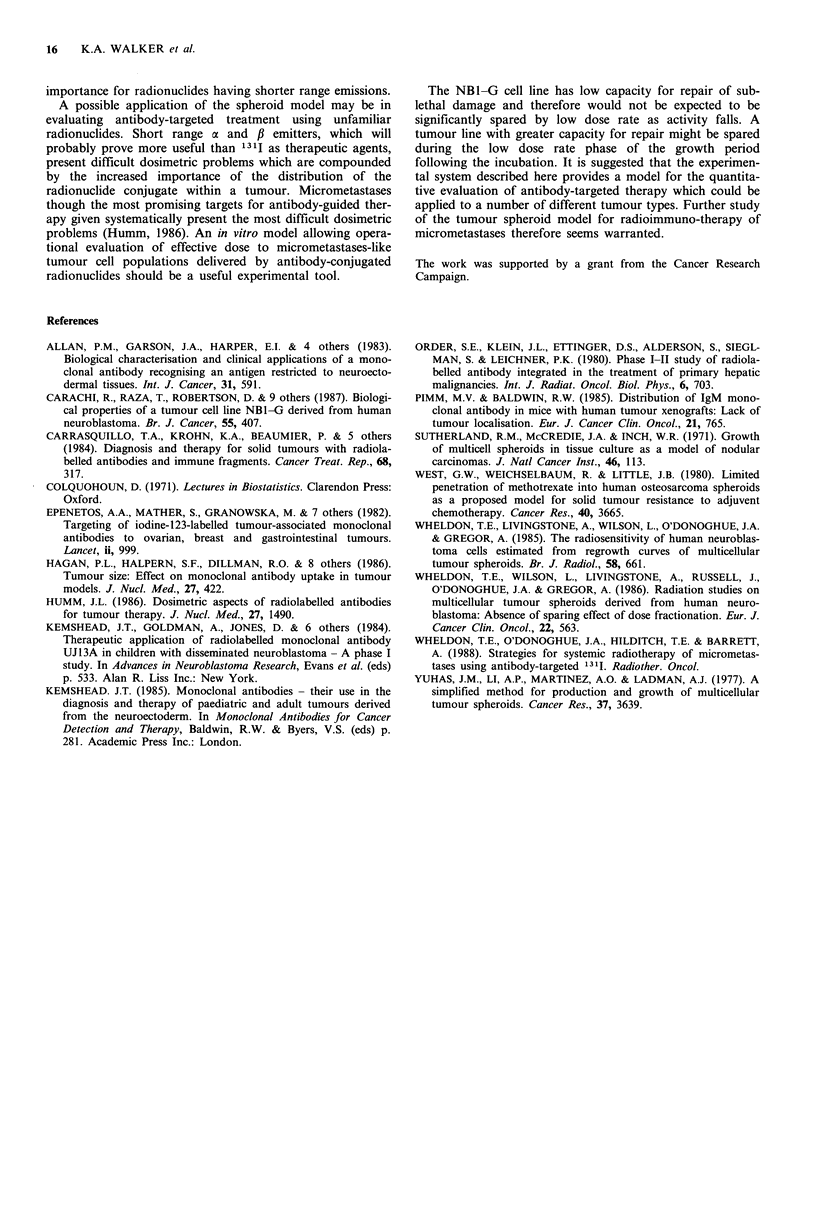# A tumour spheroid model for antibody-targeted therapy of micrometastases.

**DOI:** 10.1038/bjc.1988.152

**Published:** 1988-07

**Authors:** K. A. Walker, T. Murray, T. E. Hilditch, T. E. Wheldon, A. Gregor, I. M. Hann

**Affiliations:** Radiation Oncology Research Group, Beatson Oncology Centre, Belvidere Hospital, Glasgow, UK.

## Abstract

Human neuroblastoma cells grown as tumour spheroids were briefly incubated with a conjugate of 131I and an anti-human neuroectodermal monoclonal antibody UJ13A. Unbound 131I was removed by washing and the spheroids observed in culture conditions for up to 4 weeks. Spheroid response to irradiation was evaluated as time to reach 10x treatment volume and proportion of spheroids sterilised. Spheroid growth was found to be affected by both the activity of 131I-UJ13A and the duration of the incubation. Na[131I], 131I-HSA, 131I labelled non-specific antibody and unlabelled antibody were found to be relatively ineffective compared to 131I-UJ13A. The tumour spheroid model has applications in the evaluation of antibodies or antibody fragments and different radionuclides which may be considered for radioimmunotherapy of micrometastases.


*Br. J. Cancer* (1988), **58**, 13–16 © The Macmillan Press Ltd., 1988

### A tumour spheroid model for antibody-targeted therapy of micrometastases

K.A. Walker[1], T. Murray[2], T.E. Hilditch[2,3], T.E. Wheldon[1,3], A. Gregor[1] & I.M. Hann[4]

[1]*Radiation Oncology Research Group, Beatson Oncology Centre, Belvidere Hospital, Glasgow G31 4PG;* [2]*Department of Nuclear Medicine, Western Infirmary, Glasgow G11 6NT;* [3]*Department of Clinical Physics, University of Glasgow, 11 West Graham Street, Glasgow G4 9LF; and* [4]*Department of Haematology, Royal Hospital for Sick Children, Yorkhill, Glasgow G3 8SJ, UK.*

**Summary** Human neuroblastoma cells grown as tumour spheroids were briefly incubated with a conjugate of $^{131}$I and an anti-human neuroectodermal monoclonal antibody UJ13A. Unbound $^{131}$I was removed by washing and the spheroids observed in culture conditions for up to 4 weeks. Spheroid response to irradiation was evaluated as time to reach 10× treatment volume and proportion of spheroids sterilised. Spheroid growth was found to be affected by both the activity of $^{131}$I–UJ13A and the duration of the incubation. Na[$^{131}$I], $^{131}$I–HSA, $^{131}$I labelled non-specific antibody and unlabelled antibody were found to be relatively ineffective compared to $^{131}$I–UJ13A. The tumour spheroid model has applications in the evaluation of antibodies or antibody fragments and different radionuclides which may be considered for radioimmunotherapy of micrometastases.

The use of monoclonal antibodies conjugated to cytotoxic agents as cancer therapy is most promising for the treatment of small numbers of tumour cells, such as sub-clinical metastases (Kemshead, 1985; Wheldon *et al.*, 1988). Radioimmunotherapy using $^{131}$I conjugated to antibodies is under clinical investigation in several centres (Carrasquillo *et al.*, 1984; Kemshead *et al.*, 1984; Order *et al.*, 1980). Most experimental work is presently being performed using xenografts of human tumour in nude mice (Hagan *et al.*, 1986; Pimm & Baldwin, 1985).

Here we describe the use of an *in vitro* experimental model which is suitable for laboratory assessment of antibody-targeted radiotherapy of microscopic tumours.

Human tumour cell lines may often be grown in the form of tumour spheroids which are cellular aggregates which grow by division in the periphery (Sutherland *et al.*, 1971; Yuhas *et al.*, 1977). Tumour spheroids *in vitro* resemble micrometastases during the avascular phase of their development (West *et al.*, 1980). In these experiments we have grown a human neuroblastoma cell line (NB1–G) in the form of tumour spheroids. The cell line NB1–G is a recently established derivative of a neuroblastoma tumour whose origin, genetics, cytogenetics, antigenicity and radiobiology have been characterised (Wheldon *et al.*, 1985; Carachi *et al.*, 1987). We have used this spheroid line to evaluate the effectiveness of antibody targeted irradiation of neuroblastoma spheroids with $^{131}$I conjugated to the mouse-anti-human neuroectodermal monoclonal antibody UJ13A which has been shown to bind to human neuroblastoma cells (Allan *et al.*, 1983).

### Materials and methods

### *Monoclonal antibodies*

UJ13A, a neuroectodermal specific monoclonal antibody (Allan *et al.*, 1983; Kemshead, 1985) was kindly provided by Dr J.T. Kemshead (Imperial Cancer Research Fund). This antibody has been shown to bind to NB1–G cells by indirect immunofluorescence staining (Carachi *et al.*, 1987). In most experiments the control used was Na[$^{131}$I]. In addition, some experiments were carried out using human serum albumin (HSA) and the monoclonal antibody T2.10 (also provided by Dr J.T. Kemshead, and shown not to bind to NB1–G cells) as the controls.

Iodination of both monoclonal antibodies and HSA was carried out with Na[$^{131}$I] carrier free (IBS30 Amersham) using the Iodogen method (Epenetos *et al.*, 1982), 100 μg antibody being incubated with 37 MBq $^{131}$I. The iodination was allowed to proceed until optimal incorporation of the radiolabel had occurred. The efficiency of binding of $^{131}$I to antibody was determined by thin layer chromatography and binding efficiencies of 80–90% were routinely obtained.

### *NB1–G tumour spheroids*

A human neuroblastoma cell line NB1–G was used for the study (Wheldon *et al.*, 1985; Carachi *et al.*, 1987). Multicellular tumour spheroids were initiated by detaching cells in monolayer culture with trypsin, and placing $0.5 \times 10^6$ cells in 25 cm$^2$ flasks base coated with 1% 'Noble' agar containing 5 ml Eagles Minimum Essential medium (MEM) with 15% foetal calf serum (FCS). Alternatively $1 \times 10^6$ cells were placed in 50 ml medium in 100 ml Techne (Cambridge) spinner vessel, stirring at 40 rpm. Following 4–5 days incubation at 37°C in 5% $CO_2$, spheroids of around 200 μm in diameter were obtained.

### *Incubation of spheroids with monoclonal antibody*

Aliquots of around 40 spheroids were transferred into universal containers and incubated in 5 ml MEM containing 15% FCS and 44 mM NaI. To the spheroids was added Na[$^{131}$I] or $^{131}$I labelled antibody at the activities shown in Tables I and II. The spheroids were exposed to Na[$^{131}$I] or $^{131}$I–UJ13A for 2 h at 37°C. In a subsequent experiment, the activity of $^{131}$I–UJ13A was approximately equal in three aliquots of spheroids and the incubation time was varied (Table IV). Some preliminary work with larger spheroids was also undertaken.

Following the incubation period, the spheroids were washed by allowing them to sediment and draining off the incubation medium. This was replaced with 5 ml of fresh medium containing 44 mM NaI. This procedure was repeated 6 times in total.

Spheroids were then transferred into individual agar coated wells of a 24 well test plate (Linbro) with one plate of cells being used for each universal container. The wells contained 0.5 ml medium having 15% FCS but no NaI added. Wells were replenished at weekly intervals with 0.5 ml

Correspondence: K. Walker.
Received 2 October 1987; and in revised form 13 April 1988.

fresh medium. The growth of spheroids was quantitated by measuring spheroid volume three times weekly using an image analysis system.

Experiments were continued until 50% or more of the spheroids in each plate measured 1,000 μm in diameter or for up to 30 days (as the wells would not accommodate further aliquots of fresh medium) whichever was the sooner. By 30 days there was a clear demarcation between regrowing and non-regrowing spheroids.

### Results

The effect of radiolabelled UJ13A on NB1–G spheroid growth was determined by incubating spheroids with 2 activities of labelled monoclonal antibody and comparing this with the effect of Na[$^{131}$I]. The resulting growth curves are shown in Figure 1. A lateral displacement of the growth curves is observed at the higher activity of $^{131}$I–UJ13A. The indicated bars are ~95% confidence limits on the median of log volume measured in μm$^3$ (Colquohoun, 1971).

Table I shows the results of 3 experiments where spheroids were exposed to a range of activities of $^{131}$I–UJ13A. Results are expressed as the median time taken for spheroids to reach 10 times the original volume, and the proportion of spheroids sterilised (i.e. failed to regrow by 30 days).

Both the proportion sterilised and the time taken for the spheroids to reach 10 times their original volume can be seen to increase with increasing activity of $^{131}$I–UJ13A. At the highest activity of $^{131}$I–UJ13A used in experiments 1 and 2, more than half the spheroids were sterilised and therefore failed to reach 10 x original volume so the median time to reach this endpoint cannot be defined, consequently only the proportion sterilised data are shown for this activity. These results should be compared with the effect of Na[$^{131}$I] on spheroid growth as summarized in Table II. This shows that spheroid growth is not visibly affected by Na[$^{131}$I] at activity levels below 73 MBq. This is ~10 times the activity necessary to produce a comparable effect using $^{131}$I–UJ13A (see Table I).

**Figure 1** NB1–G spheroid regrowth curves (median log volume in μm$^3$ ± 95% confidence limits) as a function of time following incubation with the following: (×) Control; (●) 6.48 MBq Na[$^{131}$I]; (○) 5.48 MBq $^{131}$I–UJ13A; (■) 13.95 MBq $^{131}$I–UJ13A.

**Table I** Dose-response relationship of activity of $^{131}$I–UJ13A and effect on spheroid growth

| *Activity of $^{131}$I–UJ13A added (MBq)* | *Time to reach 10× treatment volume (days)* | *Proportion sterilised* |
|---|---|---|
| *Experiment 1* | | |
| 0 | 7.2 (6.8–7.8) | 0 |
| 5.48 | 9.6 (8.2–11.3) | 0.05 |
| 13.95 | 15.1 (12.4–>30) | 0.33 |
| 27.82 | – | 0.77 |
| *Experiment 2* | | |
| 0 | 9.5 (7.7–10.5) | 0 |
| 6.88 | 14 (12.0–>30) | 0.05 |
| 13.69 | 27 (15.8–>30) | 0.36 |
| 27.49 | – | 0.50 |
| *Experiment 3* | | |
| 0 | 7.0 (6.7–7.5) | 0 |
| 6.14 | 9.7 (8.6–12.7) | 0.10 |
| 16.10 | 8.1 (7.2–10.9) | 0.10 |
| 27.45 | 25 (17.4–>30) | 0.48 |

1. Median values with 95% confidence limits (Colquohoun, 1971). 2. An upper confidence limit cannot be calculated where this exceeds the duration of the experiment (30 days). 3. Medians cannot be calculated when >50% of spheroids were sterilised.

**Table II** Effect of Na[$^{131}$I] on spheroid growth

| *Activity of Na[$^{131}$I] added (MBq)* | *Time to reach 10× treatment volume (days)* | *Proportion sterilised* |
|---|---|---|
| *Experiment 1* | | |
| 0 | 7.2 (6.8–7.8) | 0 |
| 6.36 | 6.6 (6.3–6.9) | 0 |
| 13.28 | 7.1 (6.8–7.5) | 0 |
| 28.56 | 7.4 (7.2–7.9) | 0 |
| *Experiment 2* | | |
| 0 | 6.9 (5.9–8.0) | 0 |
| 21.50 | 5.3 (4.7–6.1) | 0 |
| 43.33 | 5.5 (4.9–6.3) | 0 |
| 79.55 | 6.5 (6.0–6.9) | 0 |
| *Experiment 3* | | |
| 0 | 8.0 (7.4–8.2) | 0 |
| 40.7 | 7.4 (6.9–7.9) | 0 |
| 73.3 | 9.6 (8.3–11.2) | 0 |
| 181.7 | 16.4 (13.2–21.8) | 0.14 |

1. Median values with 95% confidence limits.

Additional experiments have shown that UJ13A alone has no effect on spheroid growth, and that $^{131}$I labelled human serum albumin, and $^{131}$I labelled monoclonal antibody T2.10, which does not bind to NB1–G cells, have only the same effect on spheroid growth as Na[$^{131}$I].

In order to determine if the spheroids are receiving a greater radiation dose from $^{131}$I–UJ13A during the 2 h incubation period or during the month-long growth phase following the washing procedure, the activity of $^{131}$I–UJ13A associated with the spheroids was quantified immediately after the 6 washes in fresh medium and at time intervals during the period of spheroid growth. Results are shown in Table III. It can be seen that a much greater $^{131}$I activity remains associated with spheroids exposed to $^{131}$I–UJ13A than with those exposed to Na[$^{131}$I].

An experiment was also conducted where the activity of $^{131}$I–UJ13A added to 3 aliquots of spheroids was approximately equal, but the length of the incubation time was varied. Table IV shows that the time for spheroids to reach

**Table III**

| *Source of irradiation* | *Activity added (M Bq)* | *Activity associated per spheroid (Bq)* 1 | 2 | 3 | 4 |
|---|---|---|---|---|---|
| Na[$^{131}$I] | 52.54 | 22 | 0 | 0 | 0 |
| $^{131}$I–UJ13A | 38.11 | 520 | 66 | 53 | 75 |

Measurements were made: 1. Immediately following washing procedure; 2. After transfer to multiwell plates; 3. On day 7; 4. On day 14.

**Table IV** Comparison of effect on spheroid growth of altering exposure time to $^{131}$I–UJ13A

| *Activity of $^{131}$I–UJ13A added (M Bq)* | *Incubation time (h)* | *Time to reach 10× treatment volume (days)* | *Proportion sterilised* |
|---|---|---|---|
| 0 | 2 | 6.9 (5.9–7.9) | 0 |
| 17.28 | 1 | 6.8 (6.2–7.7) | 0 |
| 19.68 | 1.5 | 14.6 (10.4–18.2) | 0.39 |
| 18.57 | 2 | – | 0.58 |

1. Median values with 95% confidence limits; 2. Median cannot be calculated as >50% of spheroids sterilised.

10 times the treatment volume increases with increasing length of incubation time. In the case of spheroids incubated for 2 h with $^{131}$I–UJ13A, the proportion sterilised was too great to allow evaluation of the spheroid growth endpoint.

The responses of two different sizes of spheroids, 200 μm and 450 μm diameter, to incubation with varying levels of $^{131}$I–UJ13A are compared in Figure 2. It can be seen that the effect of $^{131}$I–UJ13A on the growth of both large and small spheroids is similar. It would appear from this result that both the large and the small spheroids are being irradiated to the same extent.

**Figure 2** Time taken for median volume to reach 10 times original value and estimated 95% confidence limits as a function of activity of $^{131}$I–UJ13A. (●) Spheroids with initial diam. 200 μm. (×) Spheroids with initial diam. 400 μm. *Note*: Arrow indicates that the upper confidence limits cannot be calculated as this exceeds the duration of the experiment.

### Discussion

The results demonstrate a dose-response relationship between radiation damage, evaluated as a delay in spheroid growth or proportion spheroids sterilised, and the activity of $^{131}$I associated with UJ13A at incubation. Similar activities of Na[$^{131}$I], however, were not found to affect growth of the spheroids, which would suggest that exposure to $^{131}$I–UJ13A resulted in binding of the agent to the spheroids. This was supported also by the negative findings on incubation with similar activities of $^{131}$I–HSA and $^{131}$I–$T_2$10. Studies with unlabelled UJ13A, which showed no effect on growth, would suggest that the effect on spheroid growth of $^{131}$I–UJ13A is radiation induced.

Significant effects were observed with Na[$^{131}$I] only at incubation activities of 180 M Bq where the estimated radiation dose during 2 h incubation (after allowing for rapid settling of the spheroids on the base of the incubation vessel) was in the region of 4 Gy, one half of the dose to a continuum of $^{131}$I at a concentration of 36 M Bq ml$^{-1}$.

Some attempts were made to quantify the extent to which $^{131}$I–UJ13A was bound to the spheroids. In one study where spheroids were incubated with 38 M Bq for 2 h, it was found that at the end of the incubation period, and after repeated washing, ~520 Bq of $^{131}$I were associated with each spheroid. Using theoretical estimates (Humm, 1986) of the absorbed fraction of $^{131}$I β-particles for very small spheres (~200 μm in this case) it can be calculated that this activity of $^{131}$I would deliver ~2 Gy h$^{-1}$ to each spheroid. If this binding of $^{131}$I–UJ13A took place early during the incubation stage, then clearly radiation exposure over a long period would be substantial. Subsequent measurements of spheroid associated activity during the growth phase, showed that a large proportion of the material left the spheroids rapidly. Within 2 h the estimated activity per spheroid was only 15% of the initial value (66 Bq/spheroid). A proportion of the activity appeared, however, to be firmly bound with there being ~10 Bq/spheroid at day 7 of the growth phase. Similar studies with Na[$^{131}$I] revealed that only 22 Bq were associated with each spheroid at the end of the incubation and repeated washing. This activity completely removed from the spheroids during the first 2 h following the end of the incubation period.

This result together with the observation that increasing time of exposure to $^{131}$I-UJ13A increases the effect on spheroid growth would suggest that the radiation dose is delivered partly by migration towards and binding of the radionuclide to the spheroids during the incubation period, and partly by a small amount of firmly bound residual activity being associated with each spheroid during the growth period.

It has been shown (Wheldon *et al.*, 1985) that spheroids irradiated with 2.5 Gy 4 MeV X-rays as single acute exposures take 14 days to grow to 10 × original volume. NBI–G cells grown as spheroids have little capacity for repair of sub-lethal damage (Wheldon *et al.*, 1986) therefore, the effect of protracted exposures should be similar to that of acute exposures of the same total dose. From Figure 1 it can be seen that 13.95 M Bq $^{131}$I given as $^{131}$I–UJ13A also results in the spheroids requiring 14 days to grow to 10 × original volume. In addition the measured activities of $^{131}$I associated with each spheroid would give a dose of the order of 2.5 Gy. This allows some comparison of the effect of antibody-targeted therapy and X-rays on spheroid growth in this system.

The similarity in effect of $^{131}$I–UJ13A on spheroids of 200 μm and 450 μm diameter would suggest that the cells of both large and small spheroids are being irradiated to the same extent. This is the expected result when using $^{131}$I as the radionuclide as the β particle range is sufficient to irradiate all the cells of a 450 μm spheroid even if only bound to the outer layer. The distribution of antibody-isotope conjugates within spheroids is likely to be of greater importance for radionuclides having shorter range emissions.

A possible application of the spheroid model may be in evaluating antibody-targeted treatment using unfamiliar radionuclides. Short range $\alpha$ and $\beta$ emitters, which will probably prove more useful than $^{131}I$ as therapeutic agents, present difficult dosimetric problems which are compounded by the increased importance of the distribution of the radionuclide conjugate within a tumour. Micrometastases though the most promising targets for antibody-guided therapy given systematically present the most difficult dosimetric problems (Humm, 1986). An *in vitro* model allowing operational evaluation of effective dose to micrometastases-like tumour cell populations delivered by antibody-conjugated radionuclides should be a useful experimental tool.

The NB1–G cell line has low capacity for repair of sublethal damage and therefore would not be expected to be significantly spared by low dose rate as activity falls. A tumour line with greater capacity for repair might be spared during the low dose rate phase of the growth period following the incubation. It is suggested that the experimental system described here provides a model for the quantitative evaluation of antibody-targeted therapy which could be applied to a number of different tumour types. Further study of the tumour spheroid model for radioimmuno-therapy of micrometastases therefore seems warranted.

The work was supported by a grant from the Cancer Research Campaign.

**References**

ALLAN, P.M., GARSON, J.A., HARPER, E.I. & 4 others (1983). Biological characterisation and clinical applications of a monoclonal antibody recognising an antigen restricted to neuroectodermal tissues. *Int. J. Cancer*, **31**, 591.

CARACHI, R., RAZA, T., ROBERTSON, D. & 9 others (1987). Biological properties of a tumour cell line NB1–G derived from human neuroblastoma. *Br. J. Cancer*, **55**, 407.

CARRASQUILLO, T.A., KROHN, K.A., BEAUMIER, P. & 5 others (1984). Diagnosis and therapy for solid tumours with radiolabelled antibodies and immune fragments. *Cancer Treat. Rep.*, **68**, 317.

COLQUOHOUN, D. (1971). *Lectures in Biostatistics*. Clarendon Press: Oxford.

EPENETOS, A.A., MATHER, S., GRANOWSKA, M. & 7 others (1982). Targeting of iodine-123-labelled tumour-associated monoclonal antibodies to ovarian, breast and gastrointestinal tumours. *Lancet*, **ii**, 999.

HAGAN, P.L., HALPERN, S.F., DILLMAN, R.O. & 8 others (1986). Tumour size: Effect on monoclonal antibody uptake in tumour models. *J. Nucl. Med.*, **27**, 422.

HUMM, J.L. (1986). Dosimetric aspects of radiolabelled antibodies for tumour therapy. *J. Nucl. Med.*, **27**, 1490.

KEMSHEAD, J.T., GOLDMAN, A., JONES, D. & 6 others (1984). Therapeutic application of radiolabelled monoclonal antibody UJ13A in children with disseminated neuroblastoma – A phase I study. In *Advances in Neuroblastoma Research*, Evans *et al.* (eds) p. 533. Alan R. Liss Inc.: New York.

KEMSHEAD. J.T. (1985). Monoclonal antibodies – their use in the diagnosis and therapy of paediatric and adult tumours derived from the neuroectoderm. In *Monoclonal Antibodies for Cancer Detection and Therapy*, Baldwin, R.W. & Byers, V.S. (eds) p. 281. Academic Press Inc.: London.

ORDER, S.E., KLEIN, J.L., ETTINGER, D.S., ALDERSON, S., SIEGLMAN, S. & LEICHNER, P.K. (1980). Phase I–II study of radiolabelled antibody integrated in the treatment of primary hepatic malignancies. *Int. J. Radiat. Oncol. Biol. Phys.*, **6**, 703.

PIMM, M.V. & BALDWIN, R.W. (1985). Distribution of IgM monoclonal antibody in mice with human tumour xenografts: Lack of tumour localisation. *Eur. J. Cancer Clin. Oncol.*, **21**, 765.

SUTHERLAND, R.M., McCREDIE, J.A. & INCH, W.R. (1971). Growth of multicell spheroids in tissue culture as a model of nodular carcinomas. *J. Natl Cancer Inst.*, **46**, 113.

WEST, G.W., WEICHSELBAUM, R. & LITTLE, J.B. (1980). Limited penetration of methotrexate into human osteosarcoma spheroids as a proposed model for solid tumour resistance to adjuvent chemotherapy. *Cancer Res.*, **40**, 3665.

WHELDON, T.E., LIVINGSTONE, A., WILSON, L., O'DONOGHUE, J.A. & GREGOR, A. (1985). The radiosensitivity of human neuroblastoma cells estimated from regrowth curves of multicellular tumour spheroids. *Br. J. Radiol.*, **58**, 661.

WHELDON, T.E., WILSON, L., LIVINGSTONE, A., RUSSELL, J., O'DONOGHUE, J.A. & GREGOR, A. (1986). Radiation studies on multicellular tumour spheroids derived from human neuroblastoma: Absence of sparing effect of dose fractionation. *Eur. J. Cancer Clin. Oncol.*, **22**, 563.

WHELDON, T.E., O'DONOGHUE, J.A., HILDITCH, T.E. & BARRETT, A. (1988). Strategies for systemic radiotherapy of micrometastases using antibody-targeted $^{131}I$. *Radiother. Oncol.*

YUHAS, J.M., LI, A.P., MARTINEZ, A.O. & LADMAN, A.J. (1977). A simplified method for production and growth of multicellular tumour spheroids. *Cancer Res.*, **37**, 3639.